# Effectiveness of systemic acupuncture in the control of urinary incontinence following radical prostatectomy: a randomized clinical trial

**DOI:** 10.1590/1980-220X-REEUSP-2022-0135en

**Published:** 2022-09-26

**Authors:** Mariana Ferreira Vaz Gontijo Bernardes, Luciana Regina Ferreira da Mata, Cissa Azevedo, Lívia Cristina de Resende Izidoro, Cristiana Mattos Camargos de Oliveira, Tânia Couto Machado Chianca

**Affiliations:** 1 Universidade Federal de Minas Gerais. Belo Horizonte, MG, Brazil.; 2 Universidade Federal de Jataí. Jataí, GO, Brazil.

**Keywords:** Urinary Incontinence, Lower Urinary Tract Symptoms, Pelvic Floor Disorders, Acupuncture; Prostatectomy, Clinical Nursing Research, Incontinencia Urinaria, Síntomas del Sistema Urinario Inferior, Transtornos del Suelo Pélvico, Acupuntura, Prostatectomía, Investigación en Enfermería Clínica, Incontinência Urinária, Sintomas do Trato Urinário Inferior, Distúrbios do Assoalho Pélvico, Acupuntura, Prostatectomia, Pesquisa em Enfermagem Clínica

## Abstract

**Objective:**

To evaluate the effectiveness of acupuncture associated with pelvic floor muscle training for the control of urinary incontinence following radical prostatectomy.

**Method:**

Open-label, parallel randomized clinical trial. The intervention group (n = 33) underwent eight sessions of systemic acupuncture associated with pelvic floor muscle training and the control group (n = 31) performed only pelvic floor muscle training. The outcome variable was urinary incontinence assessed by the *Pad Test* and *Daily Pad Used*, before treatment (T0), after four weeks (T1) and after eight weeks of treatment (T2). Data analysis was performed using a longitudinal model of Generalized Estimating Equations, significance level of 0.05.

**Results:**

The control group showed greater urinary loss compared to the intervention group at T1 (p = 0.006) and at T2 (p < 0.001). Both groups showed improvement in the level of urinary incontinence over time, but the improvement was greater in the intervention group (p < 0.001).

**Conclusion:**

Acupuncture associated with pelvic floor muscle training was effective in reducing urinary incontinence in prostatectomized men.

Brazilian Registry of Clinical Trials:RBR-3jm5y2

## RESUMO

**Objetivo:**

Avaliar a efetividade da acupuntura associada ao treinamento muscular do assoalho pélvico para o controle da incontinência urinária pós-prostatectomia radical.

**Método:**

Ensaio clínico aleatorizado paralelo, do tipo aberto. O grupo intervenção (n = 33) foi submetido a oito sessões de acupuntura sistêmica associada ao treinamento muscular do assoalho pélvico e o grupo controle (n = 31) somente ao treinamento muscular do assoalho pélvico. A variável desfecho foi incontinência urinária avaliada pelo *Pad Test* e *Daily Pad Used*, antes do tratamento (T0), após quatro semanas (T1) e após oito semanas de tratamento (T2).A análise de dados foi realizada por modelo longitudinal de Equações de Estimações Generalizadas, nível de significância de 0,05.

**Resultados:**

O grupo controle apresentou maior perda urinária em comparação ao grupo intervenção em T1 (p = 0,006) e em T2 (p < 0,001). Ambos os grupos apresentaram melhora no nível de incontinência urinária ao longo do tempo, porém a melhora foi maior no grupo intervenção (p < 0,001).

**Conclusão:**

A acupuntura associada ao treinamento muscular do assoalho pélvico foi efetiva para a redução da incontinência urinária em homens prostatectomizados.

Registro Brasileiro de Ensaios Clínicos:RBR-3jm5y2

## RESUMEN

**Objetivo:**

evaluar la efectividad de la acupuntura asociada al entrenamiento muscular de piso pélvico para el control de la incontinencia urinaria post-prostatectomía radical.

**Método:**

ensayo clínico aleatorizado paralelo, del tipo abierto. El grupo intervención (n = 33) fue sometido a ocho sesiones de acupuntura sistémica asociada al entrenamiento muscular de piso pélvico y el grupo control (n = 31) solamente al entrenamiento muscular de piso pélvico. La variable desfecho fue incontinencia urinaria evaluada por el *Pad Test* y *Daily Pad Used*, antes del tratamiento (T0), después de cuatro semanas (T1) y después de ocho semanas de tratamiento (T2). El análisis de datos fue realizado por modelo longitudinal de Ecuaciones de Estimaciones Generalizadas, nivel de significancia de 0,05.

**Resultados:**

el grupo control presentó mayor pérdida urinaria en comparación al grupo intervención en T1 (p = 0,006) y en T2 (p < 0,001). Ambos grupos presentaron mejor nivel de incontinencia urinaria a lo largo del tiempo, sin embargo la mejora fue mayor en el grupo intervención (p < 0,001).

**Conclusión:**

la acupuntura asociada al entrenamiento muscular de piso pélvico fue efectiva para la reducción de la incontinencia urinaria en hombres prostatectomizados.

Registro Brasileño de Ensayos Clínicos:RBR-3jm5y2

## INTRODUCTION

Prostate cancer is the second most common cancer among men, with an incidence of approximately 1.1 million per year worldwide^(1)^. The gold standard treatment for a localized tumor is radical prostatectomy (RP). Despite the benefits, post RP complications can significantly impact a man’s life and include, mainly, urinary incontinence (UI) and erectile dysfunction^(2,3)^. According to the International Continence Society (ICS), any complaint of involuntary leakage of urine can be considered UI^(4)^. The incidence of post-PR UI (PRPUI) can be 80%^(1,4)^ and can be present soon after the removal of the indwelling urinary catheter and significantly impacts the man’s quality of life^(2,5)^.

PRPUI has multifactorial causes^(3,4)^ and can be classified as stress UI and urge UI^(1,6)^. Stress UI is considered the most common PRPUI and usually occurs due to an injury to the proximal sphincter system^(1,6)^. On the other hand, urge UI is more related to detrusor overactivity^(1,6)^.

The male pelvic floor muscles help maintain urinary continence. Therefore, pelvic floor muscle strengthening restores normal function and significantly improves PRPUI^(7)^.

As for treatments for PRPUI, conservative treatment stands out as the first choice, which includes pelvic floor muscle training (PFMT) associated with behavioral changes^(8)^. The training is justified because the male pelvic floor muscles help in the maintenance of urinary continence. Therefore, pelvic floor muscle strengthening restores normal function and significantly improves PRPUI^(7)^. The changes are related to the reduction of the consumption of diuretic liquids, mainly close to bedtime; replacement of foods that promote constipation; incentive to perform physical activities; and planning of urination time intervals^(7,8)^. Other treatments are medications and surgery for artificial urethral sphincter implantation and urethral suspension^(3,9)^.

The physiological effects of PFMT can be seen after two weeks, but symptoms decrease most significantly between six and eight weeks^(10)^. PFMT requires adherence and it is a challenge to teach men about correct pelvic muscle activation^(7,11)^. However, it is considered that patient education on exercise conducted by nurses before and after surgery has a significant impact on the recovery of urinary continence^(10)^ and can be associated with other nursing interventions.

Acupuncture, for example, is a therapeutic strategy of Integrative and Complementary Practice (*PIC*) and a typical form of treatment in Traditional Chinese Medicine (TCM)^(9)^. In addition to being a valuable asset in Chinese culture, it is considered an increasingly recognized scientific tradition in urology^(5)^. It is a procedure that prioritizes curative, low-cost treatment with minimal side effects^(5)^. Therefore, its possible application in urology is diverse, including treatment of erectile dysfunction and UI^(5)^.

This science argues that all body functions are regulated to maintain a balance of *Qi*, and deviation is therefore described as disease. *Qi* is the vital energy that circulates within the individual, which maintains the organs functioning and, therefore, promotes life maintenance^(5,12)^. Thus, in the case of UI, rebalancing shall be carried out in the meridians influencing urinary continence^(5,12)^. The acupuncture points (acupoints), arranged along the channels through which the *Qi* (meridians) circulates, are stimulated with the insertion of fine needles to balance the *Qi* and, consequently, prevent or treat the disease and its symptoms^(5)^.

Acupuncture is a type of nerve stimulation technology using needles. In the context of UI and clinical practice, it can produce posterior tibial stimulation and sacral nerve balance, to play a therapeutic role that favors continence^(12,13)^. According to TCM, UI is associated with deficiency of *Qi* and *Yang* of the Spleen-Pancreas and Kidneys, and consequent humidity that compromises urethral opening and closing^(14)^. Research centers in China have conducted clinical trials to assess the safety and effectiveness of acupuncture in the management of PRPUI^(5,9)^.

It is observed that acupuncture can be used alone or associated with other TCM or conventional therapeutic procedures^(13)^. However, no studies with a robust methodology were found associating such interventions with PFMT for the treatment of PRPUI. In this context, the effect of acupuncture to reduce urine leakage among women was proven in a systematic review and meta-analysis^(8)^, but little was known about its effect and safety in men with PRPUI^(9)^.

Despite the lack of robust evidence, acupuncture is used for urge and stress UI in clinical practice^(6,9)^, to optimize muscle performance by increasing muscle tone and response speed^(14,15)^, which therefore indicates its probable effectiveness in controlling the PRPUI.

Clinical studies used acupuncture to treat UI and identified a beneficial clinical effect in improving continence ^(5,9,13)^ after at least three weeks with peak improvement at six weeks ^(8,9,12,16)^. However, no study was identified to verify the effectiveness of an acupuncture program with acupoint protocol associated with PFMT for the treatment of PRPUI.

Therefore, given the high prevalence of PRPUI and its frequent functional, emotional, and socioeconomic impacts^(2)^, it becomes important to investigate the effectiveness of non-pharmacological techniques such as acupuncture and PFMT. The possible effectiveness of the association of these techniques can improve the assistance provided to users of the Brazilian Public Health Care System in the Brazilian reality. In addition, the protocol of acupuncture points for PRPUI can guide the health professional and facilitate the implementation of *PICs* in Brazilian municipalities that do not yet have this resource.

In this context, there is an unmet clinical need and an incentive to seek effective, low-cost, and viable non-invasive treatment for the population^(7,11)^. Thus, the aim of the present study was to evaluate the effectiveness of acupuncture associated with PFMT for the control of PRPUI.

## METHOD

### Design of Study, Local and Period

This is an open-label, parallel, randomized clinical trial that followed the recommendations of the *Consolidated Standards of Reporting Trials* (CONSORT)^(17)^. The study was carried out in a hospital located in Belo Horizonte, MG, Brazil, specialized in cancer treatment. Data collection took place between April 12, 2019 and April 7, 2020.

### Population, Sample Definition and Selection Criteria

The study population consisted of 352 men undergoing RP. To estimate the sample size, the sample calculation established for clinical trials with quantitative outcomes in independent samples, and the effect of the intervention on the control of PRPUI were considered. The evaluation performed in a previous study was adopted^(18)^, with a significance level of 5%, power of 90% and variance of 67, which suggested a sample of 30.80 people. Rounding this value, in the present study, a minimum of 31 participants was established in each group. It was defined that losses during the study would imply the inclusion of new participants up to the minimum number in each group.

For screening, men who had undergone RP for less than two months and who had UI after removal of the indwelling urinary catheter were evaluated. Men with self-reported urinary leakage and daily use of pads, diapers or lining were eligible.

The inclusion criteria were having preserved cognitive, locomotor, visual, auditory, and swallowing capacity; having removed the indwelling urinary catheter between 10 and 15 days after recruitment to participate in the study; and being available to go to the institution for nine consecutive weeks. Exclusion criteria were: report of previous UI; UI with leakage of less than one gram assessed by the *Pad Test* ^(19)^; medical diagnosis of severe urinary tract infection; history of specific treatments for UI in the previous month; undergoing chemotherapy or radiotherapy; having a surgical complication. The discontinuation criteria were not attending appointments for two consecutive weeks and the need for any new surgical procedure.

Randomization into two groups was performed by a researcher external to the study, in blocks of 10 people. For each block, a sequence of random numbers was generated through the site (http://www.randomization.com/). The list with the sequence was placed in an opaque envelope, numbered and sealed by an individual external to the study team. Immediately before the first intervention session, the envelope was opened by the interventionist to identify which group the participant would belong to.

### Data Collection

The study compared two groups of participants: CG – received eight weekly sessions of PFMT; IG – received eight weekly sessions of systemic acupuncture associated with PFMT. The therapeutic regimens of the interventions implemented in the CG and IG included a protocol based on the CONSORT recommendations^(17)^ and on *Standards for Reporting Interventions in Clinical Trials of Acupuncture*.

Both groups received face-to-face monitoring to perform the PFMT, as well as guidance for continuing the exercises at home. The information written through the booklet entitled “Guidance manual on post-radical prostatectomy urinary incontinence”^(20)^ helped the researcher in the process of patient orientation and follow-up. The information in the booklet guided the weekly follow-up by emphasizing the exercise that should be performed that week. The content of the booklet included guidelines for the recognition of the pelvic muscles by the patient. The exercises were divided into six steps with description and illustration to guide their performance. The patient was evaluated weekly by the researcher who analyzed his progression and the possibility of advancement to the next stage by demonstrating his ability to perform more difficult exercises. If the patient could not, he remained in the same exercise for another week, when he was reassessed. This intervention was conducted aiming at increased pelvic floor strength, suppression of voiding urgency, and behavioral counseling.

**Figure 1:**
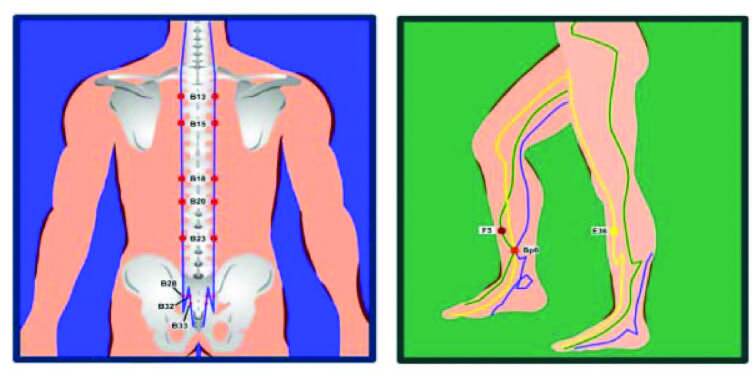
Points on the back and Liver, Spleen-Pancreas, and Stomach meridians. Belo Horizonte, MG, Brazil, 2020.

In addition to PFMT, IG participants received acupuncture weekly. The acupoint protocol was based on previous studies, the definition of PRPUI and the possible causes of prostate cancer, which led to IUPPR according to the TCM ^(5,9,14,21,22)^. Points were applied in the following order: Feishu (B13 -肺俞), Xinshu (B15 -心俞), Ganshu (B18 -肝俞), Pishu (B20 -脾俞), Shenshu (B23 -肾俞), Pangguangshu (B28 -膀胱俞), Ciliao (B32 -次), Zhongliao (B33 -中), Zusanli (E36 -足三里), Sanyinjiao (BP6 -三阴交) and Ligou (F5 - - 蠡沟) (Figure 1).

Treatment was performed with sterile and disposable acupuncture needles, size 0.25 x 30 mm, brand *Dongbang®*. Before the procedure, antisepsis was performed with cotton and 70% ethyl alcohol in the predefined locations, and then the needles were applied, remaining for 25 minutes.

**Figure 2:**
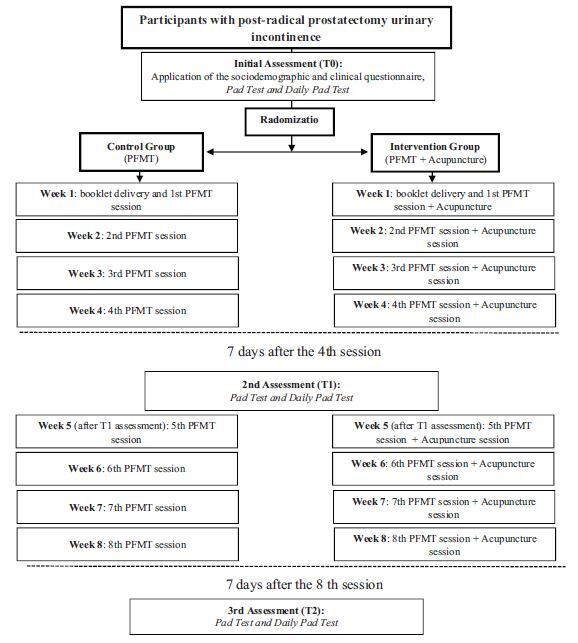
Flowchart with study outline Belo Horizonte, MG, Brazil, 2020. *PFMT: pelvic floor muscle training.

Three evaluations were performed: before treatment (T0), after four weeks (T1) and eight weeks of treatment (T2). Three instruments were applied, the *Pad Test* ^(19)^, and during the rest period, other information was obtained (sociodemographic and clinical questionnaire, *Daily Pad Used*)^(19)^. Then the men were randomized into CG and IG. All participants were evaluated by the *Pad Test* and *Daily Pad Used* in the fifth week (Test 1 – T1), before starting consultation, and in the ninth week (Test 2- T2), one week after the eighth session. The treatment flowchart and the evaluation of the study participants is shown in Figure 2.

The sociodemographic and clinical questionnaire included characterization variables and clinical conditions that may interfere with the level of PRPUI: age, education, professional status, per capita income, post-surgical time, Prostate Specific Antigen (PSA), time of indwelling urinary catheter use, prostate weight, number of comorbidities, Body Mass Index (BMI), and waist circumference^(3,23)^.

The instrument *Pad Test* of one hour, recommended and validated by the ICS, was used to quantify urinary leakage due to effort, urgency, and overflow^(19)^. To apply the *Pad test* and because it is an elderly population, the following application of the instrument was standardized: weighing the pad wrapped in a plastic bag; application of the pad close to the external urethral meatus; ingestion of 500 milliliters of water followed by rest for 15 minutes. Then, the protocol of simulation of daily activities was applied for 15 minutes (sitting down and standing up ten times, coughing ten times, picking up an object on the floor five times, washing hands for one minute, going up and down stairs for five minutes); walking for 10 minutes; and weighing of the pad after 1 hour of application. Urinary leakages are classified as: leakages of up to 1 gram (g) – insignificant; between 1.1 and 9.9 g – light leakage; between 10 and 49.9 g – moderate leakage; and above 50 g – severe leakage.

Self-reported PRPUI level was measured by the instrument *Daily Pad Used* ^(19)^. It consists of quantifying the number of diapers, pads or linings used over 24 hours. UI is classified as mild (one pad, diaper or liner), moderate (two to three) or severe (more than three).

### Data Analysis and Treatment

Data were entered into Microsoft Excel®, double-typing to test the consistency of the information. Statistical analysis was performed using statistical software *State v.* 15.0. To assess the homogeneity of the groups at T0, the normality test *Shapiro-Wilk* was used. The Student t test was adopted to one factor to verify the differences in means between the CG and IG in the variables: age, *pad test*, abdominal circumference, and time with indwelling urinary catheter. In view of non-normal distributions, non-parametric correspondents were adopted to carry out the evaluation by the Mann-Whitney test in the other variables: schooling, per capita income, *Daily Pad Used*, PSA, surgery time, BMI, number of comorbidities, prostate weight. The inferential analyses were performed considering as the primary outcome the change in urinary continence assessed by the *Pad test* of one hour, and as a secondary outcome, the UI evaluated by the *Daily Pad Used*. The generalized estimating equations (GEE) model was used to perform intra- and inter-group comparisons regarding the change in the primary outcome (*Pad test*) and the secondary outcome (*Daily Pad Used*) between CG and IG in T1 and T2 in relation to T0.

To apply the GEE in this study, a better adequacy of the outcome variable (UI) was identified with the selection of the Gaussian distribution for the family, with an identity link. When selecting the appropriate correlation structure, the autoregressive matrix was chosen, given that when there are repeated measures collected over time, the intra-individual correlation tends to decrease over time, with the AR-1 matrix being an adequate alternative. Then, robust standard errors were selected so that the estimates produced could be valid even if there was an incorrect specification of the correlation structure. The results of the GEE model were presented as regression coefficients, given that the primary outcome is continuous. The significance level adopted throughout the analysis was 5%.

### Ethical Aspects

The study was approved by the Research Ethics Committee (Opinion No. 3.043.540/2018) and registered on the Brazilian Clinical Trials Registry website (RBR-3jm5y2). The participants signed the Free and Informed Consent Form (FICF), according to Resolution 466/2012 of the National Health Council.

## RESULTS

**Figure 3:**
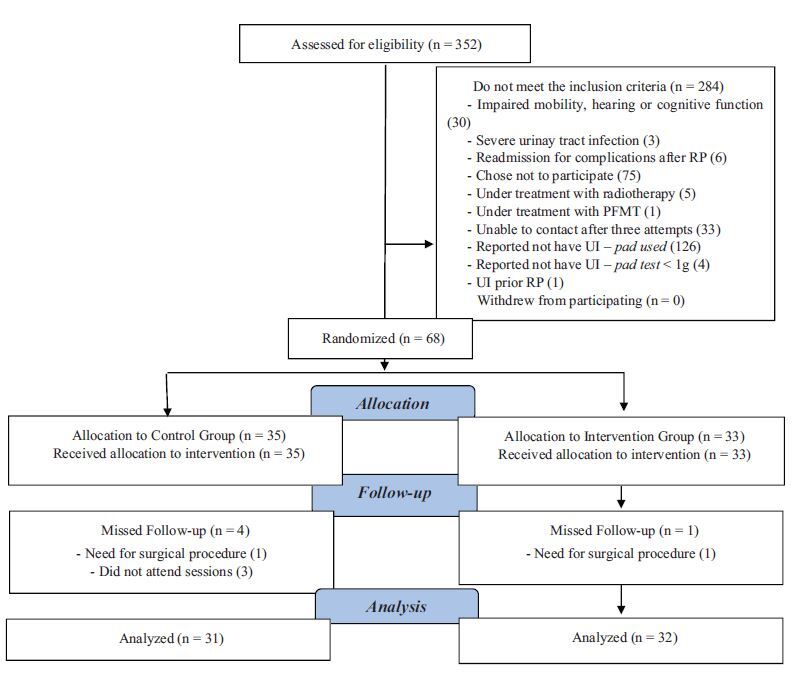
Sample tracking flowchart. Belo Horizonte, MG, Brazil, 2020. *PFMT = Pelvic floor muscle training; ‡UI = Urinary incontinence.

A total of 352 men were eligible for evaluation and, of these, 284 did not meet the inclusion criteria, with 68 being randomized into two groups. The flowchart for tracking the participants included in the study is shown in Figure 3.

The participants showed, in CG and IG, respectively, 64.52% and 71.88% retired or unemployed (p = 0.530). When groups were compared, they were homogeneous at T0 for all variables analyzed (Table 1).

**Table 1.** Characterization of participants with PRPUI in the CG (N = 31) and IG (N = 32) according to clinical variables – Belo Horizonte, MG, Brazil, 2020.

Variables	CG Mean (±SD)	IG Mean (±SD)	p value
Age (n = 63)	63.93 (7.23)	64.84 (6.40)	0.599^¶^
Education (years) (n = 63)	7.06 (3.98)	6.28 (4.60)	0.182^**^
Per capita income (n = 63)	1,069.59 (563.75)	983.45 (574.37)	0.352^**^
UI (*Pad Test*) (n = 63)	62.69 (56.81)	48.67 (55.59)	0.326^¶^
UI (*Daily Pad Used* ) (n = 61)	2.10 (0.61)	2.06 (0.56)	0.770^**^
Prostate Specific Antigen (n = 54)	41.56 (173.01)	18.17 (37.20)	0.597^**^
Time after surgery (n = 63)	30.93 (6.38)	32.75 (7.15)	0.629^**^
Time with indwelling urinary catheter (n = 63)	16.41 (3.03)	16.71 (3.62)	0.723^¶^
Body mass index (n = 63)	25.78 (4.08)	26.16 (4.04)	0.705^**^
Abdominal circumference (n = 63)	97.48 (9.02)	99.76 (9.02)	0.369^¶^
Number of comorbidities (n = 63)	2.06 (1.09)	1.65 (1.31)	0.203^**^
Prostate weight (n = 61)	46.75 (22.14)	50.31(26.84)	0.669^**^

UI = Urinary incontinence; CG = Control group; IG = Intervention group. ^¶^Student t test; ^**Mann-Whitney Test^.

The results of urinary leakage in grams evaluated by the *Pad test* and self-report by *Daily Pad Used* are shown in Table 2.

**Table 2.** Estimation of the effect of acupuncture associated with PFMT on PRPUI measured by the *Pad test* and *Daily Pad Used* based on the Generalized Estimating Equations (GEE) method – Belo Horizonte, MG, Brazil, 2022.

Group	T0		T1		T2		p-value (95%CI)		
	Mean (±SD)	95%CI	Mean (±SD)	95%CI	Mean (±SD)	95%CI	T0 – T2	T1 – T2	T0 – T1
UI (*Pad Test*)
Control Group	62.69 (±6.69)	49.59; 75.79	25.56 (±6.79)	12.23; 38.88	8.12 (±2.59)	3.03; 13.21	54.57 (32.04; 77.09)	–17.53 (–33.35; –1.71)	–37.03 (–17.96; –56.11)
							**p < 0.001**	**p = 0.024**	**p < 0.001**
Intervention Group	48.67 (±6.58)	35.77; 61.57	6.81 (±6.58)	6.086 19.711	1.33 (±0.87)	–0.39; 3.05	47.34 (24.68; 70.0)	–5.48 (–9.26; –1.70)	–41.86 (–21.16; –62.56)
							**p < 0.001**	**p = 0.002**	**p < 0.001**
P value	0.326		**0.013**		**0.013**				
UI (*Daily Pad Used*)									
Control Group	2.65 (±0.23)	2.18; 3.11	1.64 (±0.15)	1.34; 1.93	1.09 (±0.14)	0.81; 1.37	1.55 (0.95; 2.16)	–0.54 (–0.85; –0.24)	–1.01 (0.46; 1.56)
							**p < 0.001**	**p < 0.001**	**p < 0.001**
Intervention Group	2.69 (±0.19)	2.32; 3.06	0.69 (±0.14)	0.41; 0.97	0.26 (±0.11)	0.03; 0.49	2.43 (1.98; 2.88)	–0.43 (–0.71; –0.16)	–2.0 (1.56; 2.44)
							**p < 0.001**	**p < 0.001**	**p < 0.001**
P value	0.770		**<0.001**		**<0.001**				

UI = Urinary incontinence; T0 = Pre-test; T1 = Test 1 (5th week of follow-up); T2 = Test 2 (9th week of follow-up); P < 0.05 according to the Generalized Estimating Equations model.

## DISCUSSION

Although both groups showed improvement over time, when comparing groups, there was a significantly greater difference in IG. Thus, by associating acupuncture with PFMT, the response is potentiated, resulting in a greater impact on PRPUI. These findings corroborate other evidence that identified favorable results of acupuncture for UI improvement in different populations^(10,22)^.

Statistics indicate that PRPUI can regress in up to two years, but given the existence of techniques that accelerate this process, PFMT and acupuncture proved to be efficient, recognized strategies that provide significant improvement to patients^(9)^. A systematic review on the evidence of the effectiveness of PFMT in the treatment of PRPUI emphasized that the use of training, especially when associated with other therapies, contributes to the early recovery of continence^(24)^.

Study carried out to verify the effectiveness of acupuncture combined with PFMT compared to the group receiving only PFMT to treat PRPUI^(16)^ showed a significant difference in the urinary control curve (p = 0.029) between the groups after four weeks and the difference peaked at six weeks (p = 0.023). The present study confirms this finding, since the treatment strategies in both groups were shown to be effective in reducing PRPUI after four weeks.

A study evaluating the specific effects of supervised versus unsupervised PFMT showed that supervised PFMT leads to a decrease in short-term UI rates and that unsupervised PFMT had effects similar to the lack of training^(25)^. In the present study, the CG and IG participants received in-person and written guidance through the booklet, to resolve doubts about which exercises they should perform and how to perform them over the following week. It should be noted that there was satisfactory adherence of participants in CG and IG.

Meta-analysis conducted to evaluate the effect of PFMT demonstrated that this training can accelerate recovery from PRPUI in early stages and over time^(26)^. Regarding the reduction in UI severity after the eighth week of treatment, studies show that the chance of being continent after performing pelvic muscle training can be up to four times greater compared to not performing the training^(22,27)^.

The mechanism like PFMT rescues continence through the repeated voluntary contraction of the pelvic floor muscles that causes an increase in their strength and resistance^(26)^. However, the great challenge of this treatment is patient adherence^(22,27)^. Faced with this challenge, the stimulation of acupoints, which have a direct action on the organs affected in UI, can potentiate the activation of the pelvic floor muscles and potentiate the effects of PFMT for cases of low adherence to exercises^(15)^. Afferent fibers related to mechanoreceptors in the skin are activated with acupoint stimulation^(28)^.

According to the TCM, UI is mainly associated with insufficiency of *Qi* and deficiency of *yang* from the Kidney and Spleen-Pancreas. The accumulation of humidity-phlegm and humidity-heat in the bladder compromises the opening of the orifices and adequate excretion of urine^(14)^. Thus, it is necessary to tonify the Spleen-Pancreas to transport and resolve humidity-phlegm and humidity-heat, in addition to purifying it through the Lung meridian and decreasing the Liver fire^(29)^.

To regulate bladder function and treat UI, it is necessary to strengthen the Kidney meridian and nourish the essence^(14)^. The Kidney is related to the innate (pre-celestial) essence and the Spleen-Pancreas and the Stomach are related to the acquired (post-celestial) essence. By nourishing the acquired essence, Water is benefited and will strengthen the essence and potentiate urination^(29)^.

Several meridians are correlated and in some way can contribute to the energy balance that will restore continence^(28)^. Thus, treating energy imbalances in recently operated RP patients is fundamental^(5)^ to minimize PRPUI damage. In the case of PRPUI, the surgical procedure causes energy imbalance, blood loss (*Xue*) and loss of other fluids that lead to post-surgical UI^(5,14)^. In this case, in addition to surgery, one should consider the root cause for the RP, which was prostate cancer. Therefore, in addition to the Kidney and Spleen-Pancreas meridians, the Bladder, Liver, Lung and Heart meridians will often be involved^(5,21)^.

Cancer occurs due to imbalances that lead to stagnation of *Qi*, *Xue* and inflammation. The process of cancer formation is slow and accumulative, involving sedentary lifestyle, chronic diseases, and unresolved emotional factors^(21)^. The main therapeutic strategies for cancer include regulating the *Qi* and *Xue*; maintaining the unobstructed flow of the meridians; transforming phlegm-humidity; dissolving toxins^(21)^. The long-term imbalance of the *Qi* and *Xue* due to cancer leads to stasis of the Heart which compromises the metabolism and distribution of body fluids, including urinary elimination. The deficiency of *Qi* is the basis of UI and the stasis of *Xue* is the prolonged consequence. In this regard, a study^(29)^ recommends the use of acupoints ST36, SP6, and BL20 to increase the *Qi*, production and circulation of *Xue* to tonify and remove blood stasis.

Lumbosacral points such as BL23, BL28, BL32 and other *backshu*, Mu and Yuanluo points can be used for the treatment of UI, which are related to the energy imbalance that caused the UI^(29)^. The acupuncture points used in the present study were selected by the TCM theory of UI, prostate cancer, in addition to considering the action of acupuncture on somatic and autonomic innervation to the bladder. In this context, points BL32 and BL33 are often used to treat UI. They are located on the second and third sacral nerve roots, respectively^(13,14)^. The frequent use of these points occurs because deep stimulation induces the transmission of neurons to the spinal cord, which is then transmitted to the brain to adjust and provide effective urination^(13,14)^.

BL32 and BL33 belong to the Bladder meridian, and according to TCM this meridian and acupoints mainly treat diseases associated with the urinary system^(12)^. It is suggested that through these acupoints there is stimulation of the sacral nerves, detrusor, and pelvic muscles to regulate the function of the bladder, urethral sphincter and nerve innervation effector(12,14). Stimulation of these acupoints increases the resistance of the urethra and pelvic floor muscles^(14)^ making them essential for urinary continence.

Point BL23 is located at the level of L2, BL28 is located paravertebrally at the level of the second sacral foramen, and points SP6 and ST36 are located on the legs and correspond to the dermatomes on the skin of the innervation of L4-S2. Therefore, from the point of view of neuromodulation, stimulation by needles in these areas acts in the center of urination, allowing activation and effective closure of the external urethral sphincter, through somato-visceral reflexes, thus neurologically influencing the bladder function^(30)^.

To produce a protocol of points that could help other acupuncturists to treat not only the symptoms, but also the root cause of PRPUI, in addition to the points referred to as the most used (BL23, BL28, BL32, BL33, ST36, SP6)(12,14,29,30), points BL13, BL15, BL18, BL20 and LV5 were used which, according to TCM, have effects on the possible causes of prostate cancer. It was noted that the use of these lumbosacral and leg region acupoints showed clinically significant benefits in reducing PRPUI.

It should be noted that there are physiological similarities between PFMT and acupuncture, and there are suggestions that acupuncture can be considered an artificial method of muscle training^(13,15)^. It is believed that when stimulating the pelvic floor there is an increase in the maximum pressure of urethral closure, and when using lumbosacral acupoints there is a stimulus consistent with muscle contraction and that simulates the PFMT^(15)^. This way, acupuncture may facilitate reinnervation and strengthening of the pelvic floor musculature, by improving the symptoms of PRPUI. In this sense, acupuncturists report that they often stimulate various lumbosacral points and regions of the legs in the treatment of UI^(13)^.

The recruitment of participants in a single institution that involves procedures and treatment with the same surgical team and participant profile was a limitation. It is suggested that further studies are carried out in different populations to confirm the effectiveness of the intervention in different populations. Another limitation was that the sample was not stratified by the level of severity of PRPUI according to the *Pad test*. In addition, the application of interventions through standardized acupoint protocols differs from the precepts of the TCM, which recommends the individuality of the points and even different points on different days, depending on the evaluation. However, satisfactory and statistically significant results were achieved in this way, and the implementation of fixed protocols allows their replicability.

## CONCLUSION

The study confirmed the hypothesis that acupuncture in combination with PFMT potentiated the treatment of men with PRPUI in terms of reducing UI levels, compared to PFMT alone. Acupuncture associated with PFMT was considered a simple, non-invasive intervention that had clinical effectiveness for the control of urinary continence in men with PRPUI. Current evidence supports that PFMT favors continence after RP and that acupuncture further favors recovery in men undergoing RP. Therefore, the association of these techniques can improve the assistance provided to users of the Brazilian Public Health Care System. Moreover, the protocol of acupuncture points for PRPUI can guide the health professional and facilitate the implementation of *PICs* in places that do not yet have this resource. New studies with multicenter follow-up and stratified randomization by UI level should be performed to generate external validity of the findings.

## Data Availability

Considering the communication practices of Ciência Aberta, the authors inform that the data are in the SciELO Data repository under DOI: https://doi.org/10.48331/scielodata.OSCKCK.
